# Occurrence of *bla*_NDM−5_ and *bla*_NDM−29_ variants and genetic diversity among Enterobacterales in hospitalized patients in northeastern Brazil: Variability of *bla*_NDM_ in *Enterobacterales*

**DOI:** 10.1007/s42770-026-02109-1

**Published:** 2026-10-12

**Authors:** Lamartine Rodrigues Martins, Érica Maria de Oliveira, Maria Izabely Silva Pimentel, Elizabeth Maria Bispo Beltrão, Ana Catarina de Souza Lopes

**Affiliations:** Microbiology Laboratory, Tropical Medicine Unit, Center of Medical Sciences, Federal University of Pernambuco (UFPE), Recife, Brazil

**Keywords:** HAI, *bla*_NDM_, *Enterobacterales*, Multidrug-resistant, Molecular epidemiology

## Abstract

This study investigated the genetic diversity and occurrence of *bla*_NDM_ variants associated with carbapenem resistance in *Enterobacterales* obtained from colonization (rectal swabs) and infection sites in patients hospitalized in a public hospital in northeastern Brazil. A total of 58 multidrug-resistant clinical isolates were analyzed using ERIC-PCR to assess clonal relatedness, and PCR followed by amplicon sequencing to detect resistance genes (*bla*_NDM_, *bla*_KPC_, *bla*_GES_, *bla*_VIM_, and *bla*_IMP_). *Klebsiella pneumoniae* was the most frequently identified species (*n* = 28, 48.27%), followed by *Serratia marcescens* and *Proteus mirabilis*, among others. All seven *P. mirabilis* isolates were classified as XDR, as were six *K. pneumoniae* and three *S. marcescens* isolates. Amikacin (*n* = 46, 79.31% susceptible) and colistin (*n* = 37, 97.37% susceptible) exhibited the highest susceptibility rates among all antimicrobials tested against the majority of isolates. ERIC-PCR revealed multiclonal dissemination and high genetic variability among the isolates. The *bla*_KPC_ gene was the most prevalent (*n* = 32, 55.17%), followed by *bla*_NDM_ (*n* = 26, 44.83%). Among the *bla*_NDM_ allelic variants, *bla*_NDM−1_ (*n* = 12, 46.15%) was the most common, followed by *bla*_NDM−5_ (*n* = 7, 26.92%) and *bla*_NDM−29_ (*n* = 1, 3.85%). This study reports the first detection in Brazil of *bla*_NDM−5_ in *Klebsiella aerogenes*, *Citrobacter freundii*, and *Escherichia coli*, in addition to reporting the first identification of *bla*_NDM−29_ in *K. pneumoniae*. Additionally, this study gives the first report in the Americas of *bla*_NDM_ in *Klebsiella ozaenae*. These findings highlight the substantial potential for mutation and interspecies dissemination of *bla*_NDM_ variants within the Enterobacterales order.

## Introduction

Healthcare-Associated Infections (HAIs) caused by *Enterobacterales* species are a global public health concern, primarily due to the emergence of carbapenem-resistant strains, often linked to beta-lactamase production. The widespread use of beta-lactam antimicrobials has exacerbated this problem by promoting the selection and dissemination of species such as *Klebsiella pneumoniae*, which can accumulate carbapenemase-mediated resistance mechanisms, such as KPC and NDM [1–3].

The *Klebsiella pneumoniae* carbapenemase (KPC) has been one of the main resistance enzymes in *Enterobacterales*. In addition, other carbapenemases may also contribute to carbapenem resistance, including Guiana extended-spectrum (GES) β-lactamases and metallo-β-lactamases (MBLs), such as New Delhi metallo-β-lactamase (NDM), imipenemase (IMP), and Verona integron-encoded metallo-β-lactamase (VIM) [4, 5]. The presence of *Enterobacterales* that produce these enzymes, not only in patients affected by HAI, but also in those colonized, shows potential for dissemination of resistance [6, 7]. Thus, molecular surveillance of emerging carbapenemase genes is crucial.

Among MBLs, NDM is the most common in the *Enterobacterales* order [2, 4, 7–9], with 70 variants of the *bla*_NDM_ gene having been described to date [10]. The variants (*bla*_NDM−1_, *bla*_NDM−5_, and *bla*_NDM−29_) correspond to previously described alleles. Among these, the M154L substitution, which characterizes the NDM-5 variant, has been associated with greater thermal stability and increased hydrolytic activity against carbapenems and cephalosporins [11–13].

Intestinal colonization by resistant Enterobacterales may contribute to the transmission and spread of resistant bacteria, as asymptomatic carriers may act as reservoirs for the spread of resistance genes. Molecular typing techniques, such as ERIC-PCR, are essential for assessing genetic variability and clonal dissemination, which enables effective containment measures [4, 5]. Although PFGE is traditionally considered the gold standard for bacterial typing, studies have demonstrated that ERIC-PCR shows good correlation with PFGE, since it offers comparable discriminatory power while being faster and more cost-effective [14–16].

Therefore, the objective of this study is to investigate and compare the occurrence of allelic variants of the *bla*_KPC_, *bla*_NDM_, *bla*_VIM_, *bla*_IMP_ and *bla*_GES_ genes, in addition to determining the genetic variability of clinical isolates of different species of the multidrug-resistant *Enterobacterales* obtained from colonization (rectal swabs) and infection sites in patients hospitalized in a public hospital in northeastern Brazil.

## Materials and methods

### Bacterial isolates

Fifty-eight clinical isolates of *Enterobacterales* from spontaneous demand, resistant to at least one carbapenem (ertapenem, meropenem, or imipenem), from 58 patients, were collected from colonization (rectal swab) and infection sites (blood, tracheal secretion, tissue, wound secretion, urine, cavitary liquids, and catheter tip) in a public hospital in northeastern Brazil (Recife/PE), between June and December 2019 (Table 1). Biochemical identification and antimicrobial susceptibility profiles were determined using the BD Phoenix™ system with NMIC/ID-406 cards. Isolates were preserved in BHI broth with 15% glycerol at -80 °C and cultured in BHI for analysis.

**Table 1:** Clinical isolates of the *Enterobacterales* order from colonization (rectal swab) and infection in patients hospitalized in a public hospital in northeastern Brazil: sector of origin, clinical sample, carbapenem resistance genes and clonal relationship by ERIC-PCR

Clinical isolates	Sector of origin ^(a)^	Clinical sample	Resistance genes	Classification MDR or XDR	Profile of ERIC-PCR
	*Klebsiella pneumoniae*				
Kp1-A4	ICU	Blood	*bla*_KPC−2_	MDR	Kp-E1
Kp3-A4	Vascular clinic	Tissue	*bla*_KPC−2_	MDR	Kp-E2a
Kp4-A4	ICU	Tracheal secretion	*bla*_KPC−2_	MDR	Kp-E3
Kp5-A4	CCU 2	Tracheal secretion	*bla*_KPC−2_	MDR	Kp-E4c
Kp6-A4	GIE	Pleural fluid	*bla*_KPC−2_	MDR	Kp-E2b
Kp7-A4	Cardiology 2	Wound secretion	*bla*_KPC−2_	MDR	Kp-E4a
Kp9-A4	Cardiology 3	Rectal Swab	*bla*_KPC_	MDR	Kp-E4a
Kp10-A4	CCU 2	Rectal Swab	*bla*_KPC−2_	MDR	Kp-E4b
Kp14-A4	GIE	Blood	*bla*_KPC−2_	MDR	Kp-E5
Kp15-A4	GIE	Blood	*bla*_KPC−2_	MDR	Kp-E6
Kp18-A4	Cardiology 3	Rectal Swab	*bla*_NDM−29_	MDR	Kp-E7
Kp21-A4	CCU 2	Rectal Swab	*bla*_NDM−1_	MDR	Kp-E8
Kp22-A4	Cardiology 3	Urine	*bla*_NDM−1_	MDR	Kp-E9
Kp23-A4	ICU	Urine	*bla*_KPC−2_	XDR	Kp-E5
Kp26-A4	ICU	Rectal Swab	*bla*_KPC_	XDR	Kp-E5
Kp27-A4	CCU 1	Blood	*bla*_NDM_	MDR	Kp-E10
Kp29-A4	ICU	Urine	*bla*_NDM−5_	XDR	Kp-E11
Kp30-A4	ICU	Urine	*bla*_NDM−5_	XDR	Kp-E12b
Kp36-A4	GIE	Blood	*bla*_KPC−2_	MDR	Kp-E12a
Kp37-A4	CCU 2	Rectal Swab	*bla*_KPC−2_	MDR	Kp-E13
Kp38-A4	CCU 1	Rectal Swab	*bla*_KPC_	MDR	Kp-E12a
Kp39-A4	CCU 1	Rectal Swab	*bla*_KPC_	MDR	Kp-E12a
Kp40-A4	GIE	Blood	*bla*_KPC−2_	MDR	Kp-E14
Kp42-A4	ICU	Blood	*bla*_NDM−1_	MDR	Kp-E15a
Kp43-A4	ICU	Abdominal cavity fluid	*bla*_NDM_	MDR	Kp-E15a
Kp44-A4	Cardiology 2	Rectal Swab	*bla*_NDM−5_	MDR	Kp-E15b
Kp45-44	GIE	Fragment	*bla*_NDM−1_	XDR	Kp-E16
Kp46-A4	Medical clinic	Urine	*bla*_KPC−2_	XDR	Kp-E17
	*Klebsiella ozaenae*				
Koz1-A	CCU2	Rectal Swab	*bla*_NDM_	MDR	Koz-E1
Koz3-A	GIE	Blood	*bla*_KPC_	MDR	Koz-E1
Koz4-A	ICU	Abdominal cavity fluid	*bla*_KPC_	MDR	Koz-E1
	*Klebsiella aerogenes*				
Ka1-A	Cardiology 1	Rectal Swab	*bla*_NDM−5_	MDR	Ka-E1
Ka2-A	Vascular clinic	Wound secretion	*bla*_KPC_	MDR	Ka-E1
	*Proteus mirabilis*				
Pm1-A3	CCU 2	Tracheal secretion	*bla*_KPC_	XDR	Pm-E1
Pm2-A3	ICU	Catheter tip	*bla*_NDM−1_	XDR	Pm-E2
Pm3-A3	ICU	Catheter tip	*bla*_NDM_	XDR	Pm-E2
Pm4-A3	ICU	Catheter tip	*bla*_NDM−1_	XDR	Pm-E3
Pm5-A3	ICU	Tracheal secretion	*bla*_NDM_	XDR	Pm-E3
Pm8-A3	CCU 1	Catheter tip	*bla*_NDM−1_	XDR	Pm-E4
Pm9-A3	CCU 1	Tracheal secretion	*bla*_NDM−1_	XDR	Pm-E5
	*Serratia marcescens*				
Sm1-A	ICU	Blood	*bla*_KPC_	XDR	Sm-E1a
Sm2-A	CE	Blood	*bla*_KPC_	MDR	Sm-E1b
Sm3-A	ICU	Blood	*bla*_KPC_	MDR	Sm-E1b
Sm4-A	CCU	Catheter tip	*bla*_KPC_	MDR	Sm-E2a
Sm5-A	Cardiology 3	Rectal Swab	*bla*_KPC_	MDR	Sm-E1a
Sm6-A	Cardiology 3	Rectal Swab	*bla*_KPC_	XDR	Sm-E2b
Sm7-A	CCU 1	Tracheal secretion	*bla*_KPC_	MDR	Sm-E1a
Sm8-A	Vascular clinic	Catheter tip	*bla*_KPC_	XDR	Sm-E1b
	*Escherichia coli*				
Eco1-A	GIE	Urine	*bla*_KPC_	MDR	Eco-E1
Eco2-A	Cardiology 1	Rectal Swab	*bla*_NDM−5_	XDR	Eco-E2
Eco3-A	Cardiology 1	Rectal Swab	*bla*_NDM−5_	XDR	Eco-E3
	*Providencia rettgeri*				
Pre1-A	CCU 2	Rectal Swab	*bla*_NDM−1_	XDR	Pre-E1
Pre2-A	CCU 1	Tracheal secretion	*bla*_NDM−1_	XDR	Pre-E2
Pre3-A	CCU 2	Rectal Swab	*bla*_NDM_	XDR	Pre-E2
	*Providencia stuartii*				
Ps3-A	CCU 2	Urine	*bla*_NDM−1_	XDR	-
	*Providencia alcalifaciens*				
Pal1-A	ICU	Catheter tip	*bla*_NDM−1_	XDR	-
	*Pantoea agglomerans*				
Pag1-A	GIE	Blood	*bla*_KPC_	MDR	-
	*Citrobacter freundii*				
Cf1-A	CCU 1	Rectal Swab	*bla*_NDM−5_	XDR	-

a *CE* Cardiological Emergency, *GIE* General Internal Emergency, *CCU 1* Coronary Unit 1, *CCU 2* Coronary Unit 2, *ICU* Intensive Care Unit

### Antimicrobial susceptibility

The Minimum Inhibitory Concentration (MIC) was determined for the antimicrobials Amikacin (AMI), Ampicillin (AMP), Ampicillin + Sulbactam (SAM), Cefazolin (CFZ), Cefepime (FEP), Cefoxitin (FOX), Ceftazidime (CAZ), Ceftriaxone (CTX), Ciprofloxacin (CIP), Colistin (COL), Ertapenem (ETP), Gentamicin (GEN), Imipenem (IPM), Levofloxacin (LEV), Meropenem (MEM), Piperacillin + Tazobactam (TZP), Tigecylcicline (TIG) and Trimethoprim + Sulfamethoxazole (SXT) for which BD Phoenix™ 100 automated equipment was used. The MICs for colistin were determined by broth microdilution as recommended by CLSI (document M07), since automated systems are not considered reference methods for polymyxins. The results of colistin for species with intrinsic resistance (*Proteus mirabilis*, *Serratia marcescens* and *Providencia* spp.) were excluded from the susceptibility analyses and were not considered in the conclusions on the activity of colistin. Antimicrobial susceptibility testing results were initially interpreted according to the BrCAST (Brazilian Committee on Antimicrobial Susceptibility Testing) criteria, which were in effect at the time of isolate collection (2019). For the purposes of international standardization and comparability with other studies, the data were subsequently reinterpreted according to the Clinical and Laboratory Standards Institute (CLSI) guidelines, document M100-35ED (2025) [17]. The classification of isolates as MDR (multidrug-resistant) or XDR (extensively drug-resistant) followed the criteria proposed by Magiorakos et al. [18], in which MDR was defined as resistance to three or more antimicrobial classes and XDR as resistance to all tested classes except one or two.

### Extraction of total DNA

The extraction of total DNA was performed using the Wizard^®^ Genomic DNA Purification Kit (Promega), according to the manufacturer’s instructions. The DNA was quantified using Nano Drop equipment and stored at -20 °C.

### Molecular typing by the ERIC-PCR

Genetic relatedness was assessed by enterobacterial repetitive intergenic consensus-polymerase chain reaction (ERIC-PCR) using established primers [19] in 25 µL reactions containing: 100 ng/µL genomic DNA, 1.0 U of *Taq* polymerase (Promega), 2 mM dNTPs, 25 mM MgCl₂, and 10 µmol of primers. The thermal cycling protocol consisted of an initial denaturation at 92 °C for 5 min, followed by 40 cycles of denaturation at 92 °C for 1 min, annealing at 36 °C for 1 min, and extension at 72 °C for 8 min, with a final extension at 72 °C for 16 min. Each PCR run included a negative control (ultrapure water) to rule out contamination. All reactions were performed in duplicate to ensure reproducibility.

ERIC-PCR profiles were obtained by analyzing agarose gels subjected to electrophoresis using a 1.5% agarose gel in TBE buffer at a constant voltage of 100 V. A 1 Kb DNA ladder (Promega) was used as the molecular weight marker. Gels were stained with SYBR Safe (Thermo Fisher, UK) and visualized under ultraviolet light (Vilber Lourmat, Marne La Vallée, France).

The banding patterns were initially evaluated visually to assess band quality and reproducibility and subsequently analyzed using GelAnalyzer software for band detection and scoring based on the presence (coded as 1) or absence (coded as 0) of polymorphic fragments [19]. The DARwin software (Version 6.0) was used to analyze the binary matrix and generate a dendrogram based on the Jaccard coefficient and the unweighted pair group method with arithmetic mean (UPGMA). Isolates showing genetic similarity ≥ 80% were considered genetically related [19], This threshold was chosen clonal relatedness, providing a balance between sensitivity and specificity for epidemiological typing. Each individual banding pattern of an isolate was defined as its genetic profile. Isolates exhibiting 100% similarity in their genetic profiles were regarded as identical isolates (clones). Each genetically related group generated by ERIC-PCR was indicated by the letter E followed by the genetic profile number, and each bacterial species was named with an abbreviation described in Table 1.

### PCR conditions for detecting the resistance genes and amplicon sequencing

For *bla*_KPC_, *bla*_NDM_, *bla*_VIM_, *bla*_IMP_ and *bla*_GES_ PCR amplification, primers from Table 2 were used. PCR amplifications were prepared in a total volume of 25 µl per tube, containing a final concentration of 1.0 ng/µl genomic DNA, 1.0 U of Taq DNA polymerase (Promega), 0.2 mM dNTPs (Promega), 1.5 mM MgCl₂, and 1 µmol of each primer. Each amplification batch included a negative control and positive controls. For *bla*_KPC_ and *bla*_NDM_, strain P1-A4 [5] was used; for *bla*_GES_, *bla*_IMP_, and *bla*_VIM_, strains PS13-A, PS6-A, and PS4-A were used, respectively [20]. All PCR products were subjected to electrophoresis on an agarose gel containing SYBR Safe stain (Thermo Fisher, UK) and visualized under ultraviolet light (Vilber Lourmat, Marne La Vallée, France).

**Table 2:** Primers used in the PCR and sequencing of amplicons of the *bla*_KPC_, *bla*_NDM_, *bla*_VIM_, *bla*_IMP_ and *bla*_GES_ genes and primers for ERIC-PCR, in clinical isolates of the *Enterobacterales* order in patients hospitalized **in** a public hospital in northeastern Brazil

Gene	Primer	Sequence (5`- 3`)	Temp.^(a)^	Amplicon size	Reference
*bla*_KPC_	KPC-1a KPC-1b	TGTCACTGTATCGCCGTC CTCAGTGCTCTACAGAAAACC	63 °C	800	[21]
*bla*_NDM_	NDM-Fm NDM-Rm	TAAAATACCTTGAGCGGGC AAATGGAAACTGGCGACC	55 °C	439	[12]
*bla*_VIM_	VIM-F VIM-R	CAGATTGCCGATGGTGTTTGG AGG TGGGCCATTCAGCCAGA	62 °C	600	[22]
*bla*_IMP_	IMP-F IMP-R	GGAATAGAG TGG CTTAATTCTC GTGATGCGTCYCCAAYTTCACT	60 °C	232	[22]
*bla*_GES_	GES-F GES-R	GAAACCAAACGGGAGACGC CTTGACCGACAGAGGCAACT	60 °C	207	[12]
NA	ERIC-1 ERIC-2	ATGTAAGCTCCTGGGGATTAAC AAGTAAGTGACTGGGGTGAGCG	36 °C	NA	[19]

a *PCR* annealing temperature, *NA* does not apply

Isolates positive for the *bla*_NDM_ and *bla*_KPC_ genes were selected for sequencing based on their representation of different genetic profiles obtained by ERIC-PCR, different species, different clinical sites, and different resistance profiles (MDR and XDR). This approach ensured maximum genetic and epidemiological diversity among the sequenced isolates.

The amplicons were sequenced in both forward and reverse directions (bidirectional sequencing) using the Sanger method. The chromatograms were visually inspected using BioEdit software to assess read quality and to verify the absence of double peaks or overlapping signals. Bases with a Phred quality score below 20 were removed, and the sequences were assembled based on the consensus reads from both directions, resulting in sequences with an average length of 800 bp for *bla*_KPC_ and 400 bp for *bla*_NDM_. The sequences obtained were compared with reference sequences deposited in GenBank: *bla*_KPC−2_ (accession number: AY034847.1), *bla*_NDM−1_ (FN396876.1), *bla*_NDM−5_ (JN104597.1), and *bla*_NDM−29_ (MN624980.1), using the BLAST tool. Results with ≥ 99% identity and ≥ 95% coverage of the reference sequence were considered positive. Nucleotide numbering followed the convention starting from the ATG initiation codon. These substitutions resulted in the following amino acid changes: *bla*_NDM−1_ sequence (position ◊ 88 V (Valine), position ◊ 154 M (Methionine), *bla*_NDM−5_ (position ◊ 88 L (Leucine), position ◊ 154 L (Leucine). GenBank accession number (OR 360763, OR360765, OR360766).

### Approval by ethics committee

The study was approved by the Ethics Committee for Research involving Human Beings of the Federal University of Brazil (CEP/Plataforma Brazil) under number 4.459.621. The research was conducted in accordance with the Declaration of Helsinki. A declaration of participation from the participants/patients was not required, as the authors themselves did not collect personal data from patients nor did they have any contact with patients. The bacteria were provided by the microbiology laboratory of the hospital in the study.

## Results

### Origin of the different species of Enterobacterales

**Fig. 1:**
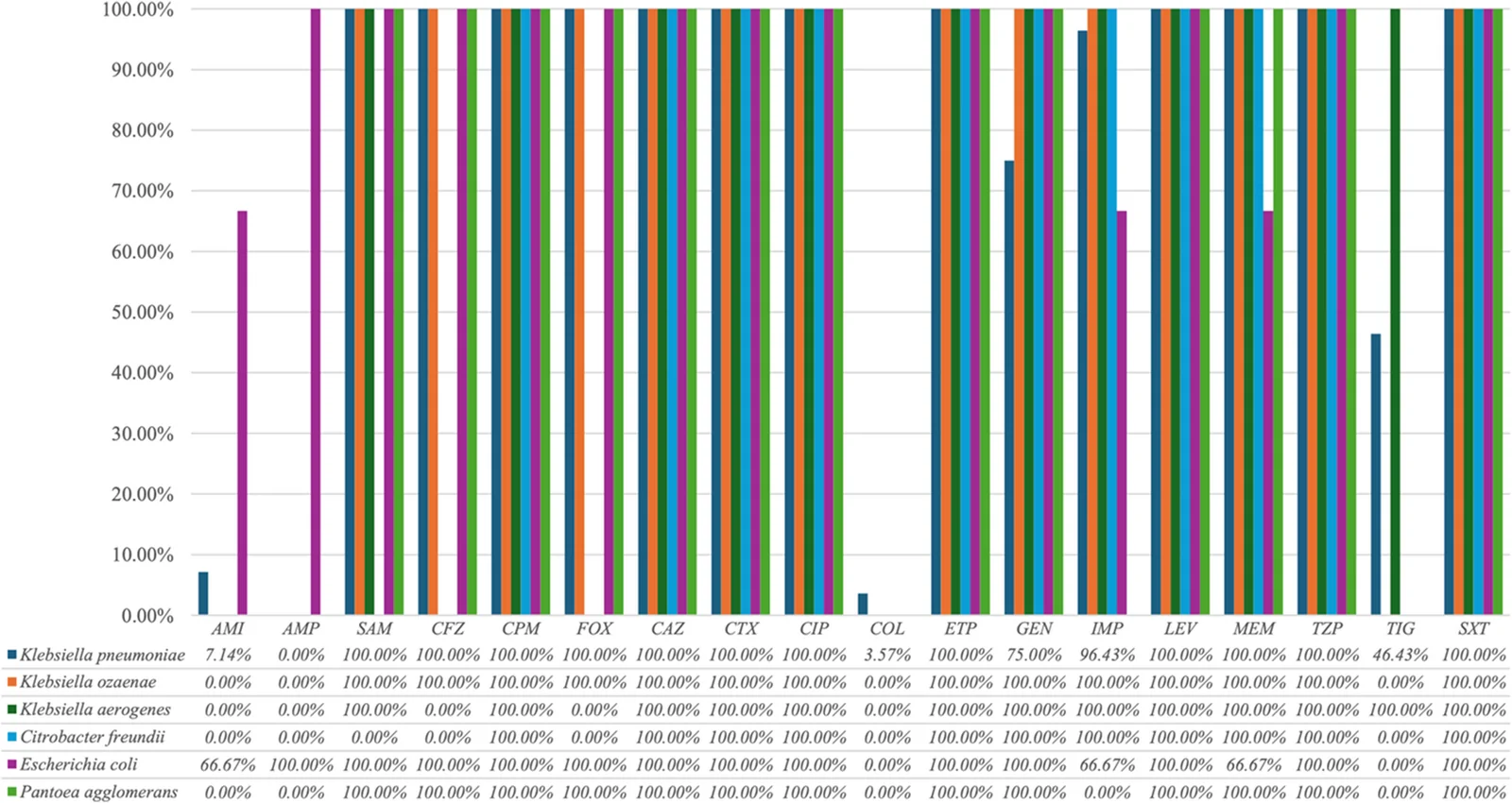
Percentage of antimicrobial resistance of different species of clinical isolates of *K. pneumoniae*, *K. ozaenae*, *K. aerogenes*, *C. freundii*, *E. coli*, and *P. agglomerans* in patients hospitalized in a public hospital in northeastern Brazil. List of intrinsic resistance profiles: *C. freundii*: ampicillin, amoxicillin, amoxicillin/clavulanate, ampicillin/sulbactam, first-generation cephalosporins, cefuroxime, and cefoxitin. *K. pneumoniae*, *Klebsiella variicola*, and *K. oxytoca*: ampicillin, amoxicillin, carbenicillin, and ticarcillin. *Klebsiella aerogenes*: ampicillin, amoxicillin/clavulanate, ampicillin/sulbactam, first-generation cephalosporins, and cephamycins (cefoxitin). *Pantoea agglomerans*: ampicillin and amoxicillin. List of antimicrobials: Amikacin (AMI), Ampicillin (AMP), Ampicillin + Sulbactam (SAM), Cefazolin (CFZ), Cefepime (FEP), Cefoxitin (FOX), Ceftazidime (CAZ), Ceftriaxone (CTX), Ciprofloxacin (CIP), Colistin (COL), Ertapenem (ETP), Gentamicin (GEN), Imipenem (IPM), Levofloxacin (LEV), Meropenem (MEM), Piperacillin + Tazobactam (TZP), Tigecycline (TIG) and Trimethoprim + Sulfamethoxazole (SXT)

**Fig. 2:**
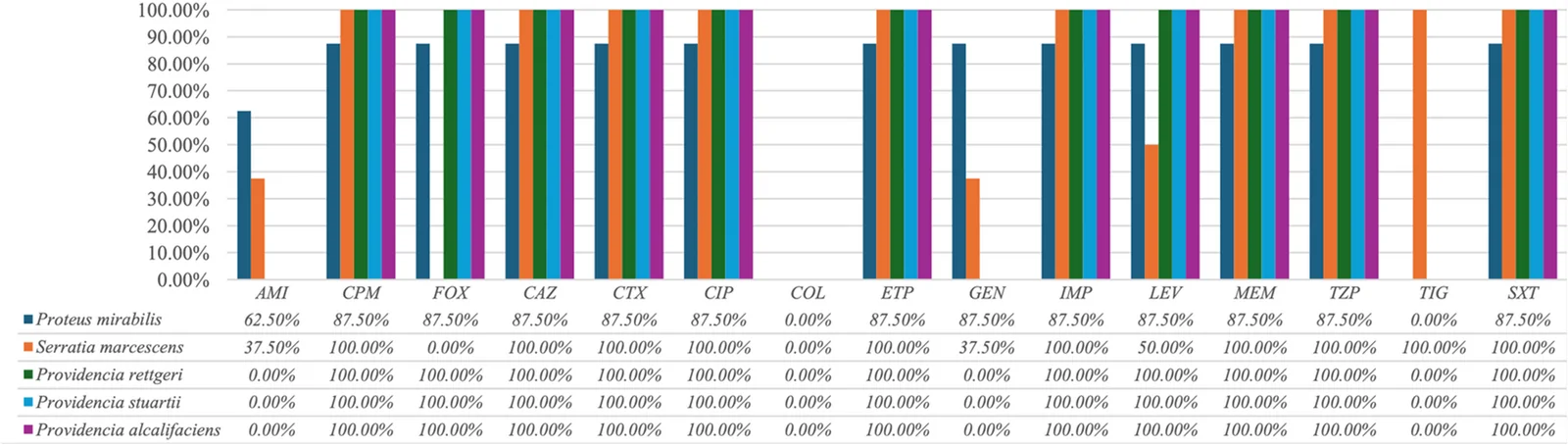
Percentage of antimicrobial resistance of different species of clinical isolates of *P*,* mirabilis*,* S. marcescens*,* P. rettgeri*,* P. stuartii*, and *P. alcalifaciens* in patients hospitalized **in** a public hospital in northeastern Brazil. List of intrinsic resistance profiles: *Providencia* spp.: ampicillin, amoxicillin, amoxicillin/clavulanate, first-generation cephalosporins, gentamicin, netilmicin, tobramycin, colistin, polymyxin B, nitrofurantoin, tetracyclines, and tigecycline. *Proteus* spp.: ampicillin, amoxicillin, first-generation cephalosporins, cefuroxime, colistin, polymyxin B, nitrofurantoin, and tetracyclines. *Serratia* spp.: ampicillin, amoxicillin, amoxicillin/clavulanate, ampicillin/sulbactam, first-generation cephalosporins, cefuroxime, cefoxitin, and colistin. List of antimicrobials: Amikacin (AMI), Ampicillin (AMP), Ampicillin + Sulbactam (SAM), Cefazolin (CFZ), Cefepime (FEP), Cefoxitin (FOX), Ceftazidime (CAZ), Ceftriaxone (CTX), Ciprofloxacin (CIP), Colistin (COL), Ertapenem (ETP), Gentamicin (GEN), Imipenem (IPM), Levofloxacin (LEV), Meropenem (MEM), Piperacillin + Tazobactam (TZP), Tigecycline (TIG) and Trimethoprim + Sulfamethoxazole (SXT)

Among the 58 carbapenem-resistant *Enterobacterales* isolates analyzed, 18 (31.03%) were from colonization and 40 (68.97%) from infections, mainly blood (*n* = 12; 20.68%), tracheal secretion, catheter tip and urine (*n* = 7; 12.06%), abdominal cavity fluid and wound secretion (*n* = 2; 3.4%), tissue, pleural fluid and fragment (*n* = 1; 1.72%). *K. pneumoniae* was the most common carbapenem-resistant species (*n* = 28; 48.27%), followed by *Serratia marcescens* (*n* = 8; 13.79%), *Proteus mirabilis* (*n* = 7; 12.06%), *Klebsiella ozaenae*, *Escherichia coli*, and *Providencia rettgeri* (*n* = 3; 5.17% each), *Klebsiella aerogenes* (*n* = 2; 3.44%), and Citrobacter *freundii*,* Pantoea agglomerans*,* Providencia alcalifaciens* and *Providencia stuartii* (*n* = 1; 1.72% each) (Table 1; Fig. 1, and Fig. 2.

### Resistance profile to antimicrobials

The resistance profile showed most isolates were resistant to all three carbapenems tested (Fig. 1), the exceptions being *K. pneumoniae* (Kp15-A4) and *P. agglomerans* (Pag1-A), sensitive to imipenem, and *E. coli* (Eco1-A), resistant only to ertapenem. Amikacin (*n* = 34, 89.47% susceptible) and colistin (*n* = 33, 97.37% susceptible) were the most effective agents against *K. pneumoniae*, *K. ozaenae*, *K. aerogenes*, *E. coli*, *C. freundii*, and P. *agglomerans.* In contrast, for inherently resistant to colistin species (*P. mirabilis*, *S. marcescens*, and *Providencia* spp.), the highest susceptibility rates were observed for amikacin (*n* = 12, 60.00%), and gentamicin (*n* = 10, 50.00%).

### ERIC-PCR typing results

**Fig. 3:**
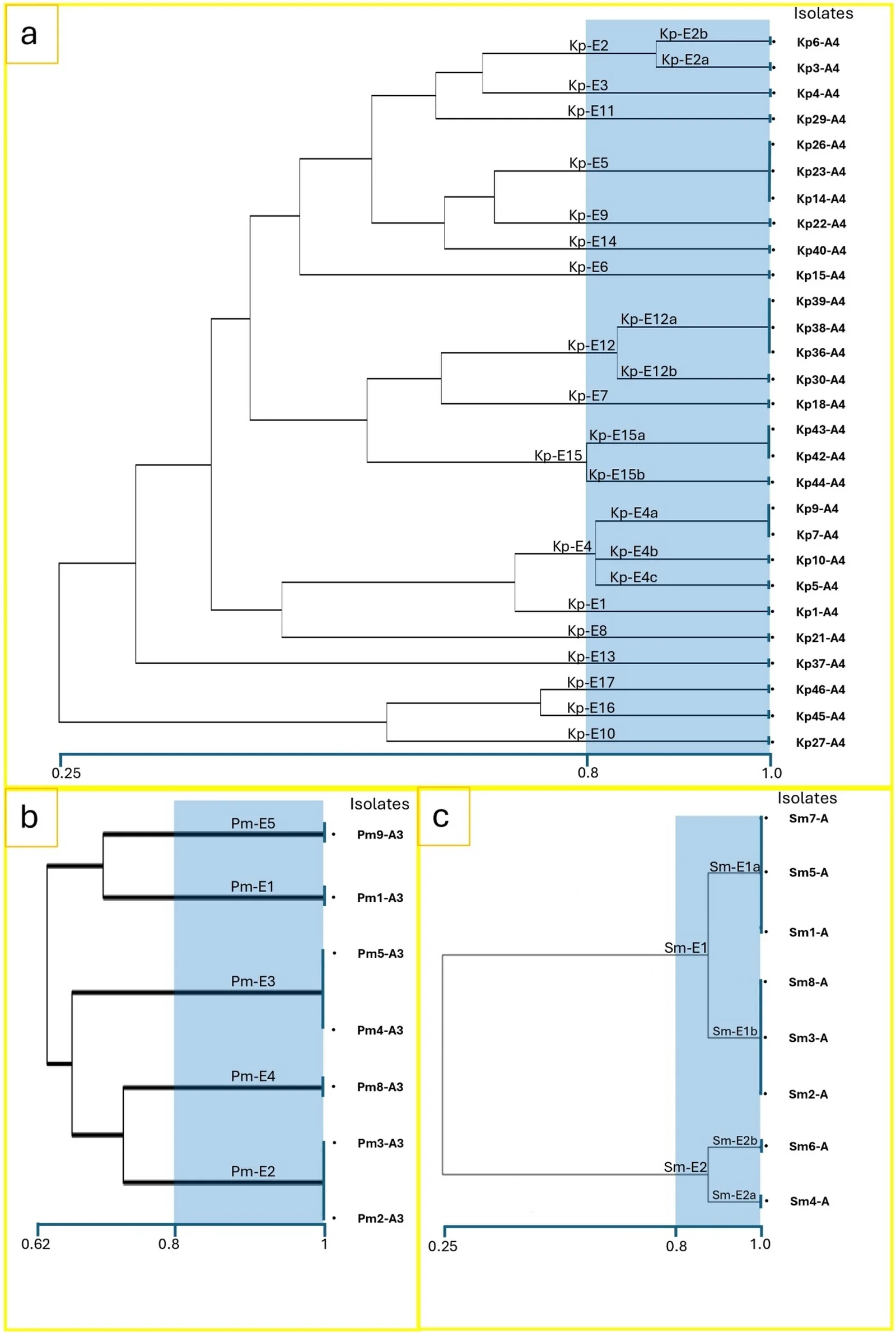
ERIC-PCR dendrograms generated using DARwin 6.0 showing the genetic relationships among (**a**) *Klebsiella pneumoniae* (Kp), (**b**) *Proteus mirabilis* (Pm), and (**c**) *Serratia marcescens* (Sm) isolates recovered from patients in a public hospital in Recife, Pernambuco, Brazil. The scale indicates the percentage of genetic similarity

ERIC-PCR analysis revealed 17 genetically related groups with 80% or higher similarity [15] after analyzing the dendrogram (Fig. 3). For isolates that showed that between 80 and 99% genetically related subgroups were considered, the letter “a” was added. They were considered the same isolate (clone) only when they exhibited 100% similarity in their genetic profile.

The 28 clinical isolates of *K. pneumoniae* in this study presented 17 genetically related groups (Fig. 3). The Kp-E5 cluster grouped three isolates of *K. pneumoniae*, two of which came from the ICU, respectively, from urine and rectal swab. The third isolate came from the internal emergency (IE) and was present in a blood sample. Thus, this shows showing that there was clonal dissemination of *K. pneumoniae* in different sectors of the hospital and clinical samples (Table 1).

The seven clinical isolates of *P. mirabilis* detected in this study showed five genetically related groups (Pm-E1, Pm-E2, Pm-E3, Pm-E4, Pm-E5). The isolates belonging to the Pm-E2 and Pm-E3 profiles came from the same hospital sector (ICU) but were present in different samples (Table 1).

The eight clinical isolates of *S. marcescens* were grouped into two genetically related groups (Sm-E1 and Sm-E2), and into four different genetic profiles (Sm-E1a, Sm-E1b, Sm-E2a, Sm-E2b) (Table 1). Two isolates (Sm4-A and Sm6-A) showed 90% genetic similarity, and came from two sectors, respectively, the Cardiac Care Unit 1 (catheter tip) and Cardiology 3 (rectal swab).

A dendrogram was not generated for these species (*K. ozaenae*,* K. aerogenes*,* E. coli* and *P. rettgeri*) because only two or three isolates were obtained per species, and the clonal relationship was analyzed based on the quantity and intensity of DNA bands generated in the agarose gel (Fig. 3). The three *K. ozaenae* isolates showed the same genetic profile (Koz-E1). Despite the clonal relationship, the isolates came from different samples (rectal swab, blood and abdominal fluid) and sectors (the General Internal Emergency “GIE” Unit and the Intensive Care Unit “ICU”). The *E. coli* isolates did not show a clonal relationship and were grouped into three different genetic profiles (Eco-E1, Eco-E2 and Eco-E3) (Fig. 3). Two isolates of *P. rettgeri* (Pre2-A and Pre3-A) showed 100% similarity and were from distinct hospital sectors, one isolate (Pre2-A) having come from tracheal secretion and the other (Pre3-A) from a rectal swab. The two *K. aerogenes* isolates showed 100% genetic similarity and came from two different hospital sectors and clinical samples (Table 1).

### Genes for carbapenemases and their variants

PCR detected carbapenem-resistant genes in all isolates, with 32 (55.17%) carrying *bla*_KPC_ and 26 (44.83%) carrying *bla*_NDM_. No isolate had both genes, and *bla*_GES_, *bla*_VIM_ and *bla*_IMP_ were not detected. The gene *bla*_KPC_ was found in all species, most frequently in *S. marcescens* (*n* = 8, 100%) and *K. pneumoniae* (*n* = 18, 64.28%). *P. mirabilis* (*n* = 6, 85.71%), *E. coli* (*n* = 2, 66.66%), and *Providencia* (*n* = 5, 100%) predominantly carried *bla*_NDM_, with *bla*_NDM−1_ (*n* = 8, 61.54%) as the most common allelic variant in this species. The presence of these genes corroborates the phenotypic profile, since these isolates showed resistance to all beta-lactams tested, including carbapenems.

The *bla*_NDM−5_ variant was detected in seven isolates from four species, comprising *K. pneumoniae* (*n* = 3, 42.86%), *K. aerogenes* (*n* = 1, 14.29%), *E. coli* (*n* = 2, 28.58%), *C. freundii* (*n* = 1, 14.29%). The *bla*_NDM−29_ variant was detected in a clinical isolate of *K. pneumoniae* (Kp18-A4). The *bla*_NDM−5_ variant presented two mutations relative to NDM-1: G262T (GTG → TTG) and A460C (ATG → CTG), resulting in the amino acid substitutions V88L (Valine → Leucine) and M154L (Methionine → Leucine), respectively. Compared with the NDM-1 reference sequence, the *bla*_NDM−29_ variant presented a single nucleotide substitution: G388A, resulting in the amino acid change D130N (Aspartic acid → Asparagine).

## Discussion

In the present study, *K. pneumoniae* was the most frequently isolated multidrug-resistant *Enterobacterales* species, while amikacin and colistin were the antimicrobials to which the isolates showed the highest susceptibility. Additionally, all *P. mirabilis* isolates were classified as extensively drug-resistant (XDR) [18]. Since its first detection, *K. pneumoniae* has received significant attention from public health authorities. Infections caused by multidrug-resistant (MDR) and XDR species of Enterobacterales have become major challenges for healthcare institutions due to limited antibiotic options and high mortality rates [23–25].

The isolates of the genus Providencia also presented as XDR (*n* = 5, 100%). Species such as *P. mirabilis*,* S. marcescens* and *Providencia* spp. have intrinsic resistance to several antimicrobials, such as ampicillin, cephalosporins, polymyxins and tigecycline [5, 26], thus making therapeutic options more restricted, thereby making infections caused by these bacteria even more serious for the patients affected [26]. In the present study, amikacin, and gentamicin showed the highest susceptibility rates among the antimicrobials tested against *Providencia* spp. isolates (Fig. 2).

Intestinal colonization of hospitalized patients by MDR *Enterobacterales* is associated with increased infections and mortality. In this paper, 31.03% (*n* = 18) of the isolates distributed among seven different species came from rectal swabs for surveillance research, in which patients who had been colonized by these bacteria were from ICUs or wards. Studies show that long hospital stays can favor colonization by these bacteria and increase transmission within the hospital environment [27, 28]. Therefore, individuals colonized by these resistant bacteria must be identified quickly and accurately so that measures to prevent and control infection can be implemented.

In this study, a high variability of clones was detected in different hospital sectors (Table 1; Fig. 3.a). This polyclonal spread of *K. pneumoniae-*carrying resistance genes has already been described in hospitals in several countries [29–31], as well as in Brazil, where this research group has previously reported the circulation of KPC-2-producing clones [5] and the association of *bla*_NDM−1_ with *bla*_KPC−2_ and AME genes in *Enterobacterales* isolates [7].

Considering the high prevalence of multidrug-resistant *K. pneumoniae*, it is important to determine the clonal relationship between the isolates by tracing the clonal profile circulating in the institution, so that it is possible to adopt appropriate containment measures.

**Fig. 4:**
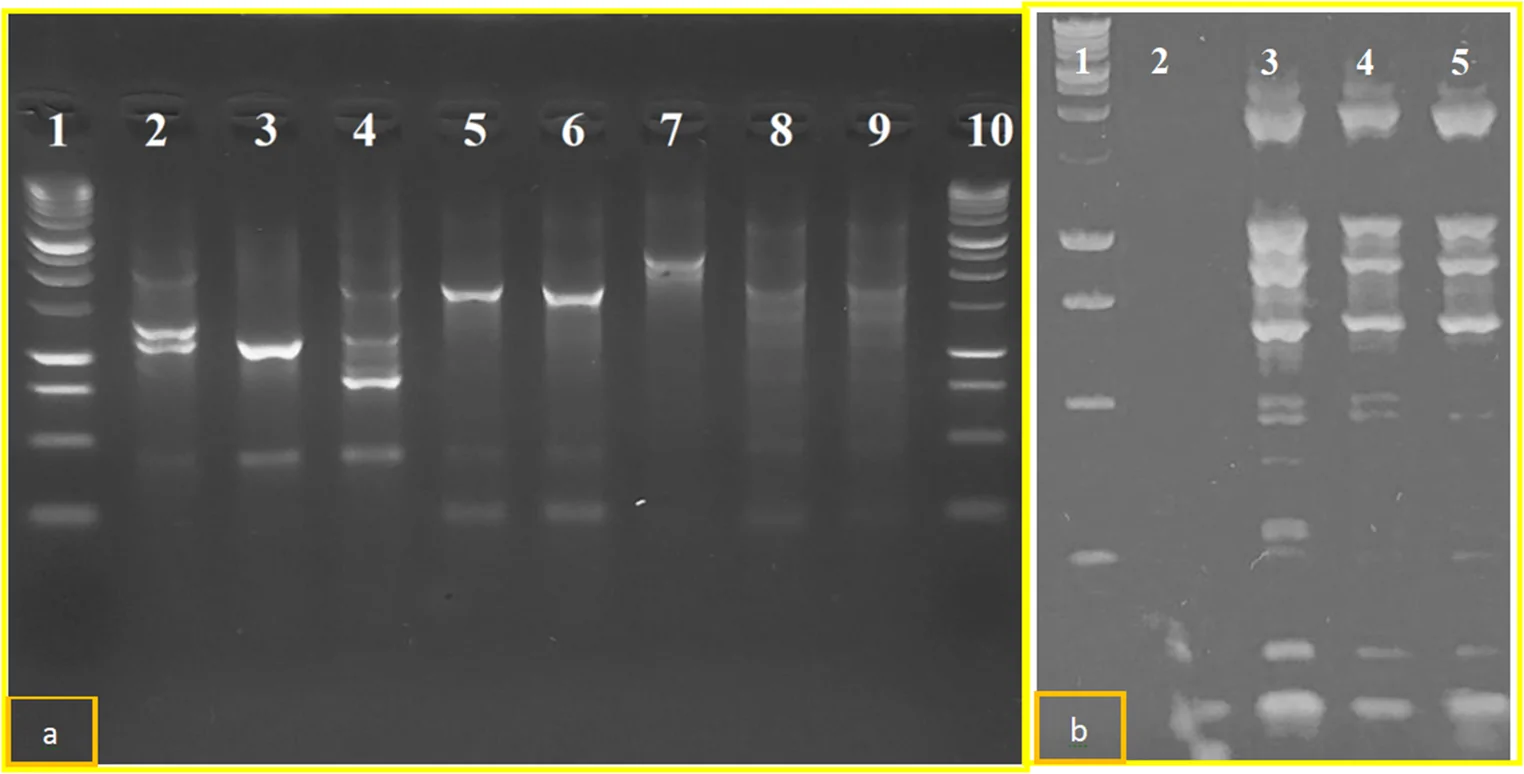
Electrophoresis in 1.5% agarose gel, showing the clonal relationship of clinical isolates of the species. (**a**) *Escherichia coli*,* Klebsiella aerogenes* and *Providencia rettgeri.* Lanes1 and 10: 1Kb molecular weight marker. Lanes 2 to 4: *E. coli* isolates (Eco1, Eco2 and Eco3). Lanes 5 and 6: *K. aerogenes* isolates (Ka1 and Ka2). Lanes 7 to 9: *P. rettgeri* isolates (Pre1, Pre2 and Pre3). (**b**) Lanes 1: 1Kb molecular weight marker. Lanes 2: Negative Control. Lanes 3 a 5: *K. ozaenae* isolates (Koz1-A, Koz3-A Koz4-A)

The analysis of genetic variability by ERIC-PCR of the other species of *Enterobacterales*, analyzed in this paper, also showed genetic diversity and polyclonal dissemination. Some clones were identified in different hospital sectors and, in some cases, were recovered from different specimen types (Table 1; Fig. 4). For example, two *K. aerogenes* isolates exhibited clonal genetic profiles but carried different carbapenemase genes: isolate Ka1-A, recovered from a rectal swab, carried *bla*_NDM−5_, whereas isolate Ka2-A, recovered from wound secretion, carried *bla*_KPC_. ERIC-PCR analysis revealed that *K. pneumoniae* (17 clusters) and *P. mirabilis* (5 clusters) showed the greatest clonal diversity (Fig. 3). These two species also exhibited the greatest variety of resistance profiles to the antimicrobials tested.

Genes that encode carbapenemases have been increasingly reported in species of the *Enterobacterales* in recent years worldwide [2, 9, 11], including in Brazil [1, 3, 4, 32]. In the present study, the majority of *Enterobacterales* clinical isolates carried the *bla*_KPC−2_ gene, mainly *Klebsiella* sp. and all *S. marcescens* isolates. The dissemination of *bla*_KPC_ occurs mainly due to its transfer between species through plasmids and transposons, in particular Tn4401 [33], but also in non-Tn4401 elements, such as NTEKPC-IId [34].

In the present study, the *bla*_KPC−2_ gene was detected in an isolate of *P. agglomerans* (*n* = 1) (Table 1), coming from blood. *P. agglomerans* has been described as an opportunistic pathogen associated with infections resulting from penetrating injuries caused by plant materials, such as thorns and wood splinters [35], and also as an HAI agent, causing bloodstream infections and pneumonia, with the *bla*_OXA181_ gene recently being identified as mediating resistance to carbapenems [36–38]. In Brazil, *bla*_KPC_ in *P. agglomerans* has also been detected [39].

Another resistance beta-lactam enzyme that has been spreading worldwide since 2009 is NDM [40]. It has received attention due to its rapid global dissemination, its evolution and its high levels of resistance to β-lactam antibiotics [41]. Since its first detection in a *K. pneumoniae* isolate from a urinary tract infection in India, it has been described mainly in the genus *Klebsiella*, but also in *P. mirabilis*, *Providencia* spp. and *S. marcescens*, with *bla*_NDM−1_ being the most common variant [2, 4, 7–9].

In this study, an isolate of *K. ozaenae* (*n* = 1, 33,33%) (Koz1-A) was detected carrying the *bla*_NDM_ gene (Table 1), this being the first report in the Americas. Other studies detected the *bla*_NDM−1_ variant in China and *bla*_NDM−5_ variant in India [42, 43]. The *bla*_NDM−5_ variant was first described in the United Kingdom in 2011, in *E. coli* recovered from a patient who had been previously admitted to a medical center in India [11]. NDM-5 differs from NDM-1 by two amino acid substitutions at positions 88 (Val → Leu) and 154 (Met → Leu), which cause reduced susceptibility to cephalosporins and carbapenems [11]. Studies show the detection of *bla*_NDM−5_ in *K. aerogenes*, from different clinical samples in China and Japan [44–46], and in *E. coli*, mainly in China [47–50]. Another study shows the presence of the *bla*_NDM−5_ variant in *C. freundii* in China from intestinal samples of fish [51]. These data show that mutations and substitutions in NDM amino acids can have a significant impact on the enzymatic activity against carbapenems, making antibiotic therapy difficult.

In Brazil, the first detection of the *bla*_NDM−5_ variant was reported in 2023 by this research group. This was found in two *K. pneumoniae* isolates obtained from a rectal swab (K32-A4) and urine (K34-A4) from patients hospitalized in a hospital in Recife, Pernambuco, Brazil [3]. Both isolates also carried the *bla*_KPC−2_ gene, and sequence analysis revealed two specific mutations (G262T and A460C) [3].

In the present study, the *bla*_NDM−5_ variant was detected in one isolate of *K. aerogenes*, one isolate of *C. freundii*, two isolates of *E. coli* and three isolates of *K. pneumoniae*, this being the first detection of this variant in *K. aerogenes*, *C. freundii* and *E. coli* in Brazil. These data are worrying, as they show an increase in the frequency of another variant of the *bla*_NDM_ gene present in different species of *Enterobacterales*, which can spread worldwide.

Another variant detected in this study was *bla*_NDM−29_. The gene sequence of this variant was first described in a *K. pneumoniae* isolate and submitted to NCBI by researchers from Saint Petersburg, Russia [52]. Soon after, namely in 2019, there was a detailed report of an NDM-29-producing *E. coli* isolate isolated from the bile of a patient in China [53]. These two studies showed that there is no difference in the hydrolytic spectrum of NDM-1 and NDM-29 enzymes in terms of susceptibility to carbapenems. In this research, the *bla*_NDM−29_ variant was detected in an isolate of *K. pneumoniae* (Kp18-A4) from a colonization sample. This strain was resistant to all β-lactams tested, while remaining susceptible to amikacin, colistin, and tigecycline. This was the first detection of this variant in Brazil.

A limitation of this study is that all *Enterobacterales* isolates were collected from a single tertiary hospital, which may limit the generalizability of the findings. The genetic profiles and resistance patterns observed could be specific to this hospital’s environment and patient population. Despite this, valuable insights into the genetic characteristics and resistance profiles of Enterobacterales in a tertiary care setting are provided.

Mutations and variability detected in *bla*_NDM_ gene (*bla*_NDM−1_, *bla*_NDM−5_ and *bla*_NDM−29_) in this study, in addition to the *bla*_KPC−2_ variant, in different species of *Enterobacterales*, demonstrates the potential of these bacterial species to suffer mutation in these resistance genes, which can increase the MIC of antimicrobials and make antibiotic therapy difficult. Furthermore, there is a risk of dissemination of genetic resistance mechanisms among species within this bacterial order which may facilitate their global spread. Therefore, there is a strong need for public health authorities to set up systems and mechanisms to monitor whether these potential situations are developing; to characterize their clonal profiles; and to draw up and implement effective control measures.

Variability detected in the *bla*_NDM_ gene (*bla*_NDM−1_, *bla*_NDM−5_ and *bla*_NDM−29_) in this study, in addition to the *bla*_KPC−2_ variant, in different species of *Enterobacterales*, evidences the genetic diversity of these bacteria and the circulation of resistance alleles in this hospital. These factors highlight the substantial potential for mutation and interspecies dissemination of *bla*_NDM_ variants within the Enterobacterales order.

## Data Availability

The data underlying this article are presented in the article.
